# Improvement in l-ornithine production from mannitol via transcriptome-guided genetic engineering in *Corynebacterium glutamicum*

**DOI:** 10.1186/s13068-022-02198-8

**Published:** 2022-09-19

**Authors:** Libin Nie, Yutong He, Lirong Hu, Xiangdong Zhu, Xiaoyu Wu, Bin Zhang

**Affiliations:** 1 Jiangxi Engineering Laboratory for the Development and Utilization of Agricultural Microbial Resources, Jiangxi Agricultural University, Nanchang, 330045 China; 2 College of Bioscience and Bioengineering, Jiangxi Agricultural University, Nanchang, 330045 China

**Keywords:** *Corynebacterium glutamicum*, l-Ornithine, Transcriptome analysis, Genetic engineering, Microbial fermentation

## Abstract

**Background:**

l-Ornithine is an important medicinal intermediate that is mainly produced by microbial fermentation using glucose as the substrate. To avoid competition with human food resources, there is an urgent need to explore alternative carbon sources for l-ornithine production. In a previous study, we constructed an engineered strain, *Corynebacterium glutamicum* MTL13, which produces 54.56 g/L of l-ornithine from mannitol. However, compared with the titers produced using glucose as a substrate, the results are insufficient, and further improvement is required.

**Results:**

In this study, comparative transcriptome profiling of MTL01 cultivated with glucose or mannitol was performed to identify novel targets for engineering l-ornithine-producing strains. Guided by the transcriptome profiling results, we modulated the expression of *qsuR* (encoding a LysR-type regulator QsuR), *prpC* (encoding 2-methylcitrate synthase PrpC), *pdxR* (encoding a MocR-type regulator PdxR), *acnR* (encoding a TetR-type transcriptional regulator AcnR), *CGS9114_RS08985* (encoding a hypothetical protein), and *CGS9114_RS09730* (encoding a TetR/AcrR family transcriptional regulator), thereby generating the engineered strain MTL25 that can produce l-ornithine at a titer of 93.6 g/L, representing a 71.6% increase as compared with the parent strain MTL13 and the highest l-ornithine titer reported so far for *C. glutamicum*.

**Conclusions:**

This study provides novel indirect genetic targets for enhancing l-ornithine accumulation on mannitol and lays a solid foundation for the biosynthesis of l-ornithine from marine macroalgae, which is farmed globally as a promising alternative feedstock.

**Supplementary Information:**

The online version contains supplementary material available at 10.1186/s13068-022-02198-8.

## Background

Microbial fermentation of valuable chemicals from renewable resources is a promising route for achieving carbon neutrality and a sustainable economy. l-Ornithine is an important organic acid that has been widely used as a pharmaceutical intermediate for the treatment of complex liver diseases [1]. The demand for l-ornithine is growing steadily owing to the increasing number of people affected by liver diseases worldwide. The high market demand has made the development of efficient l-ornithine production routes a hot research topic. Microbial fermentation is an efficient approach for l-ornithine production, but it requires high-performance recombinant strains to minimize costs. Currently, *Corynebacterium glutamicum*, a Gram-positive soil bacterium, has been used to produce l-ornithine on a commercial scale and is the most competitive l-ornithine producer [2]. With the completion of *C. glutamicum* sequencing and resolution of metabolic pathways, persistent efforts have been made to construct l-ornithine-producing strains over the past few years [3]. These strain breeding strategies can be summarized as follows: metabolic evolution [4, 5], blocking competitive metabolic pathways [6–8], overexpression of key genes [9, 10], removal of feedback inhibition in the l-ornithine biosynthesis pathway [11, 12], increasing the supplementation of precursors by modifying the glycolysis and tricarboxylic acid cycle (TCA cycle) [13], increasing NADPH availability [10, 14, 15], and unblocking the secretion system by overexpression of *lysE* [7]. Notably, deletion of *argF*, *argR*, and *ncgl2228*, as well as overexpression of *CsgapC* and *BsrocG*, resulted in the engineered strain *C. glutamicum* KBJ11, which exerted the best l-ornithine production performance of 88.26 g/L and a yield of 0.414 g/g glucose [14]. However, this superior performance was obtained using glucose as a substrate, which competes with human food resources.

The food crisis caused by the growing population has prompted researchers to explore more sustainable feedstocks to produce l-ornithine. Various carbon sources such as xylose [13], molasses [16], sucrose [17], glycerol [18], and arabinose [19] have been used as second-generation biomass feedstocks to produce l-ornithine. However, the obtained l-ornithine production titers have not exceeded 20 g/L, which severely limits the application of these carbon sources. Therefore, attempts have been made to expand the application of third-generation biomass feedstock for microbial fermentation production of l-ornithine. For instance, we previously developed an engineered strain MTL13 that could produce 54.56 g/L of l-ornithine from mannitol, a sugar alcohol that can be easily extracted from ocean-farmed macroalgae globally [20, 21]. This l-ornithine technical parameters is significantly higher than that obtained using second-generation biomass, but there is still room for improvement compared with the performance using glucose as a carbon source. However, microbial metabolism and regulation are complex processes, rational engineering targets are almost exhausted, and rational metabolic engineering strategies have little effect on the improvement of strain performance owing to insufficient comprehensive analysis.

Recently, the rapid development of omics techniques has been widely used for revealing metabolic regulatory networks and directing metabolic modification [5, 22–24]. Transcriptome analysis is commonly used to identify new target genes for optimizing the performance of industrial strains [25, 26]. For instance, transcriptome analysis was used to elucidate the molecular mechanism by which the addition of betaine resulted in increased l-leucine production [27]. Using transcriptome analysis, novel targets related to l-leucine biosynthesis were identified and applied to further enhance l-leucine production [27]. Transcriptome analysis allows us to understand genome-wide differences in gene expression and identify potential target genes for the optimization of l-ornithine-producing strains. Therefore, we performed a comparative transcriptome analysis between mannitol and glucose as the sole carbon sources for MTL01 fermentation. In this study, we identified six novel targets that exerted a positive effect on l-ornithine biosynthesis. Single, double, and triple manipulations of these genes were used to promote the conversion of mannitol to l-ornithine.

## Results and discussion

### Transcriptome changes in response to mannitol or glucose

**Fig. 1:**
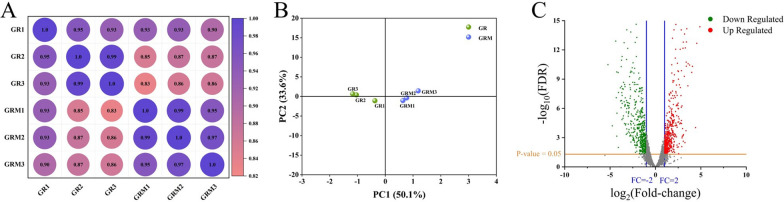
Quality assessment of transcription samples GRM and GR. **A** Correlation analysis of samples. The number represents the correlation coefficient, and the closer the correlation coefficient is to 1, the higher the similarity of gene expression between samples. **B** Principal component analysis (PCA) of samples. **C** Volcano plots of gene expression difference. Red dots represent significantly upregulated genes, green dots represent significantly down-regulated genes, and gray dots represent non-significantly altered genes, the transverse lines in orange represent *p* = 0.05, the vertical lines in blue represent fold-change (FC) =  − 2/2

In a previous study, we focused on finding non-competitive raw materials to produce l-ornithine and constructed the engineered strain MTL13 by deletion of *mtlR* and overexpression of the *mtlTD* operon and *pfkB*, which could produce 54.56 g/L of l-ornithine from mannitol [21]. However, although the yield of l-ornithine obtained using mannitol is higher than that of second-generation carbon feedstocks, it is still inferior to the production titer obtained using glucose [14]. To further improve the performance of strain MTL13, transcriptome profiling techniques, powerful instruments for mechanism analysis, were employed to identify new indirect targets for gene manipulation. Thus, we performed Illumina RNA-seq analysis for the engineered strain *C. glutamicum* MTL01 using mannitol (GRM) or glucose (GR) as the sole carbon source to investigate the changes in global gene expression. The correlation coefficient of biological samples within the group was close to 1 in the correlation analysis score plot, and there was a slightly clear separation between the groups in the principal component analysis (PCA) diagram, which indicated favorable repeatability in the group and a clear distinction between the GRM and GR groups (Fig. 1A, B). The software DESeq2 was used to analyze and identify differentially expressed genes (DEGs) between the GRM and GR groups, which identified 2939 genes in Illumina RNA-seq data. Among them, the transcription levels of 1002 genes, including 471 upregulated genes and 531 down-regulated genes, were significantly altered if the parameter was set to twofold (Fig. 1C). These results indicated that the transcriptional data had high quality and reliability. Nearly one-third of the genes showed altered expression, suggesting that culture of strain MTL01 with different carbon sources has a remarkable effect on its physiological metabolism. In theory, these DEGs are probably involved in mannitol metabolism and are potential genetic modification targets for increasing the yield of l-ornithine from mannitol.

### Comparative enrichment analysis of top 20 DEGs

**Fig. 2:**
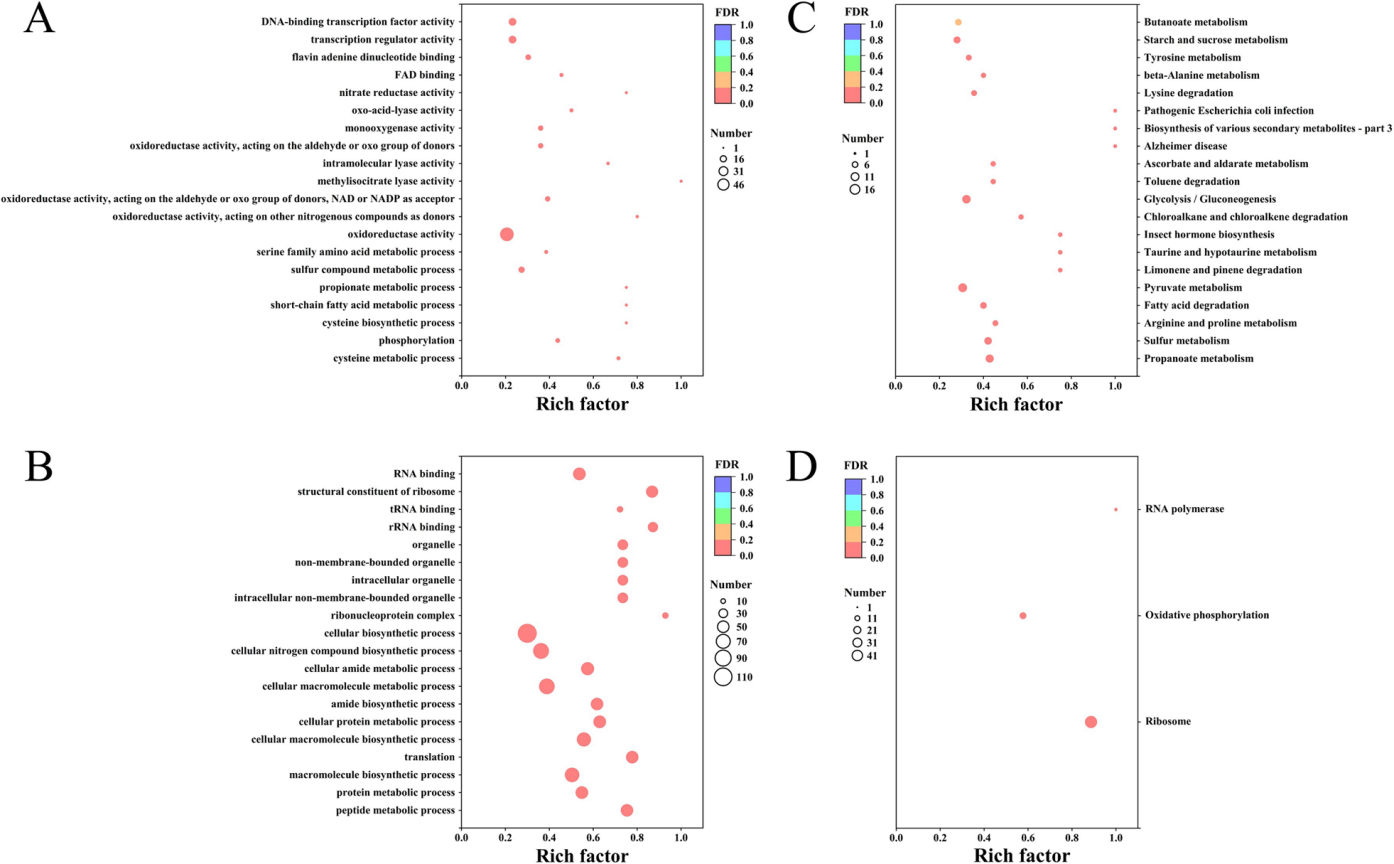
GO and KEGG enrichment analysis of top 20 differentially expressed genes (DEGs) between GRM and GR groups. The size and color of the dot represents number and degree of enrichment of DEGs in the GO and KEGG pathway. Rich factor: ratio of differentially expressed annotated genes in a GO term or a KEGG pathway. **A** GO terms of top 20 upregulated DEGs. **B** GO terms of top 20 down-regulated DEGs. **C** KEGG pathway enrichment of top 20 upregulated DEGs. **D** KEGG pathway enrichment of down-regulated DEGs

To further investigate the potential mechanisms related to mannitol metabolism, the top 20 DEGs were classified using gene ontology (GO) and Kyoto Encyclopedia of Genes and Genomes (KEGG) annotation analyses. Theoretically, DEGs with superior expression changes are more strongly correlated with mannitol metabolism. From the GO function enrichment analysis, it was found that the upregulated genes were highly capable of oxidoreductase activity, transcription regulator activity, and DNA-binding transcription factor activity, while the down-regulated genes can be enriched followed by cellular nitrogen compound biosynthetic process and cellular macromolecule metabolic process (Fig. 2A, B). In the KEGG enrichment analysis, it was found that propanoate and pyruvate metabolism pathways were significantly enriched and upregulated, while the ribosome, oxidative, and RNA polymerase were down-regulated (Fig. 2C, D). Eight upregulated genes were involved in the TCA cycle, and the methyl citric acid cycle indicated that *C. glutamicum* MTL01 cultured with mannitol had a strong energy metabolism. In particular, *CGS9114_RS13295*, identified as *prpC*, encoding a bifunctional enzyme that participates in the methyl citric acid cycle and TCA cycle, showed the highest expression change of 20.88-fold (Table 1). Subsequently, we summarized the differentially expressed transcriptional regulators that frequently respond to changes in the composition of the medium and regulate the expression of multiple genes. A total of 17 transcriptional regulatory factors belonging to diverse families, including LysR, TetR/AcrR, and IclR, were identified of which seven were upregulated and ten were down-regulated in the GRM groups (Table 2). In addition, the expression of genes, including *pobA*, *catA*, *catB*, *benK*, *benE*, *pheA1*, *dadA*, *nagX*, *pcaG*, *aroC*, *aroB*, *aroK*, and *aroP*, related to aromatic compound metabolic pathways were also changed, which suggested that the utilization of mannitol inhibited the biosynthesis pathway of aromatic metabolites in *C. glutamicum* (Table 3).

**Table 1:** Genes associated with the tricarboxylic acid cycle and propionic acid metabolism

Gene	Description	Log2(FC)	Padjust	Regulated type
*CGS9114_RS13295* (*prpC*)	Bifunctional 2-methylcitrate synthase/citrate synthase	4.38	1.11E−13	Up
*CGS9114_RS02685*	Methylisocitrate lyase	4.11	9.13E−13	Up
*CGS9114_RS13300*	Methylisocitrate lyase	3.79	2.47E−09	Up
*CGS9114_RS02680*	MmgE/PrpD family protein	3.48	1.12E−16	Up
*CGS9114_RS01580*	NAD-dependent succinate-semialdehyde dehydrogenase	3.41	2.51E−19	Up
*CGS9114_RS02690*	Bifunctional 2-methylcitrate synthase/citrate synthase	3.28	8.54E−10	Up
*CGS9114_RS13305*	MmgE/PrpD family protein	2.72	8.41E−06	Up
*CGS9114_RS06605* (*acnR*)	TetR/AcrR family transcriptional regulator	− 2.28	2.28E−03	Down

**Table 2:** The transcription factors in differentially expressed genes in MTL13 with mannitol versus glucose

Gene	Description	Log2(FC)	Regulated type
*CGS9114_RS05160* (*qsuR*)	LysR family transcriptional regulator	3.17	Up
*CGS9114_RS02635*	LysR family transcriptional regulator	3.01	Up
*CGS9114_RS12125*	FadR family transcriptional regulator	3.17	Up
*CGS9114_RS03825*	IclR family transcriptional regulator	2.43	Up
*CGS9114_RS11915*	Winged helix-turn-helix transcriptional regulator	2.23	Up
*CGS9114_RS04045*	IclR family transcriptional regulator	2.29	Up
*CGS9114_RS01690*	AraC family transcriptional regulator	2.21	Up
*CGS9114_RS09730*	TetR/AcrR family transcriptional regulator	− 4.67	Down
*CGS9114_RS13900*	TetR/AcrR family transcriptional regulator	− 2.48	Down
*CGS9114_RS06605*	TetR/AcrR family transcriptional regulator	− 2.28	Down
*CGS9114_RS15050*	Winged helix-turn-helix transcriptional regulator	− 1.77	Down
*CGS9114_RS06050*	YebC/PmpR family DNA-binding transcriptional regulator	− 1.36	Down
CGS9114_RS12885 (*pdxR*)	PLP-dependent aminotransferase family protein	− 1.32	Down
CGS9114_RS15035	Heavy metal-responsive transcriptional regulator	− 1.32	Down
CGS9114_RS12980	WhiB family transcriptional regulator	− 1.24	Down
CGS9114_RS11310	YlxR family protein	− 1.20	Down
CGS9114_RS09095	GntR family transcriptional regulator	− 1.04	Down

**Table 3:** Genes related to synthesis and catabolism of aromatic compounds

Gene	Description	Log2(FC)	Gene name	Regulated type
*CGS9114_RS14035*	4-Hydroxybenzoate 3-monooxygenase	3.21	*pobA*	Up
*CGS9114_RS08655*	Catechol 1%2C2-dioxygenase	2.66	*catA*	Up
*CGS9114_RS08625*	Aromatic acid/H + symport family MFS transporter	2.47	*benK*	Up
*CGS9114_RS08620*	Benzoate/H ( +) symporter BenE family transporter	2.00	*benE*	Up
*CGS9114_RS13620*	Chorismate mutase	1.44	*pheA1*	Up
*CGS9114_RS03770*	FAD-binding oxidoreductase	1.15	*dadA*	Up
*CGS9114_RS03835*	3-Hydroxybenzoate 6-hydroxylase	1.13	*nagX*	Up
*CGS9114_RS08680*	Protocatechuate 3%2C4-dioxygenase subunit alpha	1.09	*pcaG*	Up
*CGS9114_RS08660*	Benzoate degradation	1.07	*catB*	Up
*CGS9114_RS06235*	Chorismate synthase	− 1.43	*aroC*	Down
*CGS9114_RS06245*	3-Dehydroquinate synthase	− 1.41	*aroB*	Down
*CGS9114_RS06240*	Shikimate kinase	− 1.31	*aroK*	Down
*CGS9114_RS03560*	Aromatic amino acid transport protein AroP	− 1.14	*aroP*	Down

### Improvement of l-ornithine production by manipulating novel transcriptome analysis-guided targets using a promoter or terminator insertion approach

**Fig. 3:**
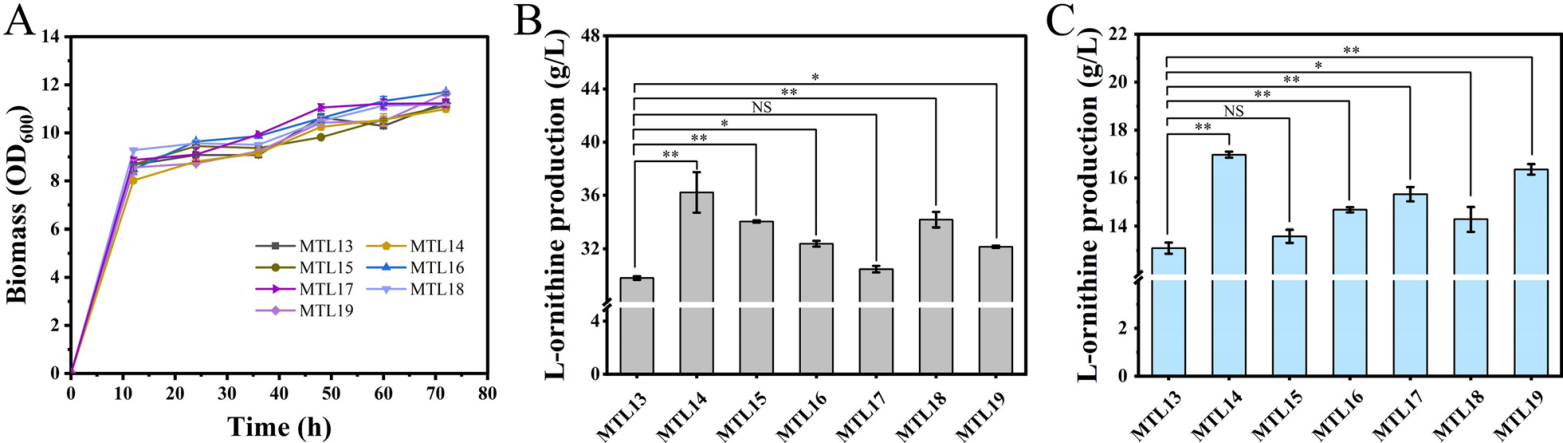
Shake-flask evolution of the engineered strain MTL13, MTL14, MTL15, MTL16, MTL17, MTL18, MTL19. Cell growth (**A**), l-ornithine formation of 48 h (**B**) and 72 h (**C**) of those strains. Results of standard deviations present in three individual experiments. *p < 0.05, **p < 0.01 and NS indicated no significant difference

From the transcriptome results, these DEGs were rarely directly involved in the biosynthesis pathway of l-ornithine; instead, the top 20 DEGs were closely related to propionic acid metabolism, the TCA cycle, aromatic metabolic pathways, and transcription regulation. The direct gene target in the biosynthesis pathway of l-ornithine was not available in the transcriptome analysis. Thus, we selected six targets based on the principle of superior multiple fold gene expression changes, highly relevant to the l-ornithine biosynthesis pathway, and preferentially selected transcription factors for gene manipulation in strain MTL13. Among them, *prpC*, encoding a bifunctional enzyme that exhibits citrate synthase and 2-methylcitric acid synthase activity, was upregulated by 20.88-fold in GRM groups [28, 29]. It is rational to speculate that overexpression of *gltA* encoding citrate synthase enhances carbon metabolic flow towards the TCA cycle [30], which promotes the conversion of mannitol to l-ornithine. AcnR is a TetR-type transcriptional regulator that inhibits the expression of *acn*, which encodes an aconitase responsible for converting citric acid to isocitric acid in the biosynthesis pathway of l-ornithine [31, 32]. QsuR, encoded by *CGS9114_RS05160*, is a LysR family transcriptional regulator that positively controls the expression of the *qsuABCD* operon and plays an important role in the catabolism of aromatic compounds [33]. We assumed that significant changes in the expression of *qsuABCD* channeled more metabolic flow from quinic acid and shikimic acid to the β-ketoadipate pathway, which provides more energy for mannitol metabolism [34]. Next, because the transcription factors can simultaneously regulate the expression levels of multiple genes, *CGS9114_RS09730* and *pdxR* [35] were selected for gene operation. Finally, *CGS9114_RS08985* (upregulated 7.51-fold) was selected to represent the hypothetical protein, which contained several genes capable of high-fold expression changes in the GRM group. Therefore, inspired by the semi-rational analysis results, six genes, including *prpC* (encoding 2-methylcitrate synthase PrpC, upregulated 20.88-fold), *CGS9114_RS08985* (encoding a hypothesis protein, upregulated 7.51-fold), *acnR* (encoding a TetR-type transcriptional regulator AcnR, down-regulated 3.86-fold), *CGS9114_RS09730* (encoding a TetR/AcrR family transcriptional regulator, down-regulated 24.46-fold), *pdxR* (encoding a MocR-type regulator PdxR, down-regulated 1.5-fold), and *qsuR* (encoding a LysR-type regulator QsuR, upregulated 9.02-fold), were selected as potential targets for individual gene manipulation in the parent strain MTL13. To test whether these gene modifications are beneficial for mannitol metabolism and l-ornithine biosynthesis, six recombinant strains were constructed and named MTL14 (P_sod_ promoter was inserted in the upstream region of *qsuR*), MTL15 (P_sod_ promoter was inserted in the upstream region of *prpC*), MTL16 (P_sod_ promoter was inserted in the upstream region of *CGS9114_RS08985*), MTL17 (T terminator was inserted in the upstream region of *acnR*), MTL18 (T terminator was inserted in the upstream region of *CGS9114_RS09730*), and MTL19 (T terminator was inserted in the upstream region of *pdxR*), following the principle of overexpressing upregulated genes and attenuating down-regulated genes. The results of shake-flask fermentation showed that these strains share identical cell growth, and the OD_600_ was distributed at approximately 11 at 72 h (Fig. 3A). It can be concluded that these genes are not necessary for cell growth and that their genetic manipulation is feasible. In addition, during 72 h of cultivation, the engineered strains MTL14, MTL15, MTL16, MTL17, MTL18, and MTL19 produced l-ornithine at titers of 36.20, 34.03, 32.38, 30.47, 34.17, and 32.16 g/L, representing a 21.4%, 14.0%, 8.6%, 2.2%, 14.6%, and 7.8% increase, respectively, compared to the production titer obtained by the parent strain MTL13 (29.81 g/L) (Fig. 3B and Table 4). Simultaneously, the titers at 48 h fermentation exhibited a consistent trend with that at 72 h fermentation, which suggested that manipulating these six genes exerts a positive effect on l-ornithine accumulation from mannitol (Fig. 3C). Among them, strain MTL14 displayed the highest l-ornithine production titer, indicating that overexpression of *qsuR* (encoding a LysR-type regulator QsuR) is beneficial for the biosynthesis of l-ornithine (Table 4). This result is consistent with our hypothesis that enhanced catabolism of aromatic compounds in the β-ketoadipate pathway, thereby degrading acetyl-CoA and succinyl-CoA, provides more energy and a precursor for the conversion of mannitol to l-ornithine. In addition, the engineered strain MTL15 displayed the second highest l-ornithine production titer, indicating that overexpression of *prpC* (encoding 2-methylcitrate synthase PrpC) accelerated the methyl citric acid cycle and that the TCA cycle can promote l-ornithine accumulation. Associated with the TCA cycle, interruption of *acnR* (encoding a TetR-type transcriptional regulator AcnR) in the engineered strain MTL17 removed the inhibition of *acn* (encoding aconitase ACN), thus improving the supply of isocitric acid for l-ornithine biosynthesis, which complements the genetic target we previously not manipulated in the TCA cycle. Although *CGS9114_RS08985* (encoding a hypothesis protein) and *CGS9114_RS09730* (encoding a TetR/AcrR family transcriptional regulator) were not clearly annotated, these results indicate that it is feasible to improve strain performance using a transcriptome-guided genetic engineering approach.

**Table 4:** The recombinant *C. glutamicum* for l-ornithine production in this study

Strains	l-Ornithine titer (g/L)	Residual mannitol concentration (g/L)	Cell biomass (OD_600_)	Yield (g/g mannitol)
MTL13	29.81 ± 0.13	11.68 ± 1.05	11.28 ± 0.08	0.43
MTL14	36.20 ± 1.52	11.31 ± 0.66	10.99 ± 0.03	0.52
MTL15	34.03 ± 0.08	12.06 ± 0.13	11.12 ± 0.09	0.50
MTL16	32.38 ± 0.22	10.87 ± 0.92	11.70 ± 0.01	0.46
MTL17	30.47 ± 0.24	10.95 ± 0.34	11.22 ± 0.17	0.44
MTL18	34.17 ± 0.57	10.69 ± 0.04	11.17 ± 0.01	0.49
MTL19	32.16 ± 0.08	10.39 ± 0.06	11.65 ± 0.11	0.46
MTL20	34.32 ± 0.61	10.95 ± 0.10	11.12 ± 0.18	0.49
MTL21	37.29 ± 0.31	11.47 ± 0.37	11.55 ± 0.09	0.54
MTL22	31.93 ± 0.44	10.41 ± 0.17	11.99 ± 0.12	0.45
MTL23	32.02 ± 0.18	11.41 ± 0.16	11.61 ± 0.05	0.46
MTL24	38.36 ± 1.30	10.79 ± 0.53	11.26 ± 0.03	0.55
MTL25	42.01 ± 0.50	10.24 ± 1.90	11.20 ± 0.33	0.60

### Combination of the novel targets and their effect on L‑ornithine production from mannitol

**Fig. 4:**
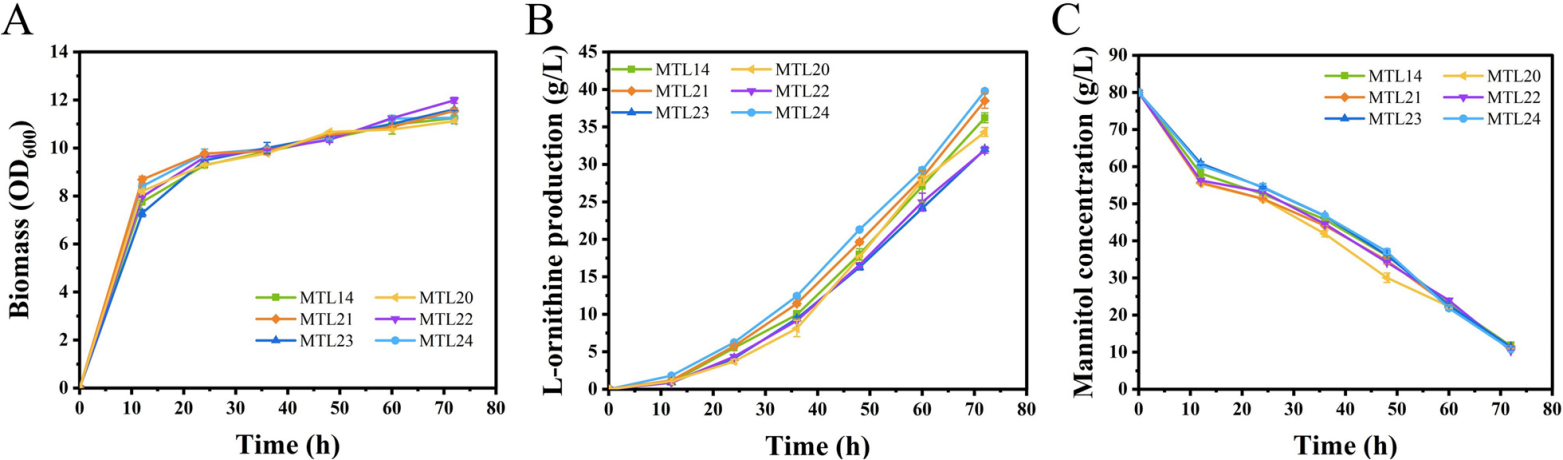
Effects of *prpC* and *CGS9114_RS08985* overexpression and *acnR*, CGS9114_RS09730 and *pdxR* attenuation basing *C. glutamicum* MTL14 on l-ornithine fermentation. l-ornithine production (**A**), cell growth (**B**) and Mannitol residue (**C**) of strains *C. glutamicum* MTL14, MTL20, MTL21, MTL22, MTL23, MTL24 during fermented in batch fermentation with flasks were compared. Results of standard deviations present in three individual experiments

It has been proven that combined metabolic engineering is an efficient strategy for improving the titer of target products [36]. To further improve the yield of l-ornithine, five candidate gene modulations, including overexpression of *prpC* (encoding 2-methylcitrate synthase PrpC) and *CGS9114_RS08985* (encoding a hypothesis protein) and attenuation of the expression of *acnR* (encoding a TetR-type transcriptional regulator AcnR), *CGS9114_RS09730* (encoding a TetR/AcrR family transcriptional regulator), and *pdxR* (encoding a MocR-type regulator PdxR), were individually engineered in the strain MTL14, thereby generating strains MTL20, MTL21, MTL22, MTL23, and MTL24, respectively. Next, we tested the performance of those strains by using shake-flask fermentation, which indicated that the engineered strains MTL20, MTL21, MTL22, MTL23, and MTL24 produced 34.31, 38.49, 31.92, 32.01, and 39.77 g/L of l-ornithine during 72 h of cultivation, respectively (Fig. 4A and Table 4). The engineered strains MTL21 and MTL24 produced 6.2% and 9.8% more l-ornithine, respectively, than the parent strain MTL14 (36.20 g/L), indicating that overexpression of *CGS9114_RS08985* (encoding a hypothesis protein) and attenuation of *pdxR* (encoding a MocR-type regulator PdxR) can synergistically promote l-ornithine biosynthesis with overexpression of *qsuR* (encoding a LysR-type regulator QsuR). In contrast, the production titers of the engineered strains MTL20, MTL22, and MTL23 were lower than that of the control strain MTL14, which suggested that enhancing the TCA cycle is not an effective strategy for improving l-ornithine production after manipulation of *qsuR* (encoding a LysR-type regulator QsuR). QsuR may be associated with the TCA cycle because it controls the decomposition of quinic and shikimic acids into acetyl-CoA and succinyl-CoA. Simultaneously, the OD_600_ values and mannitol concentration did not differ between these strains, indicating that these modulations did not affect the normal physiological metabolism of *C. glutamicum* (Fig. 4B, C, Table 4). This is consistent with the results of the previous rounds of single modulations.

**Fig. 5:**
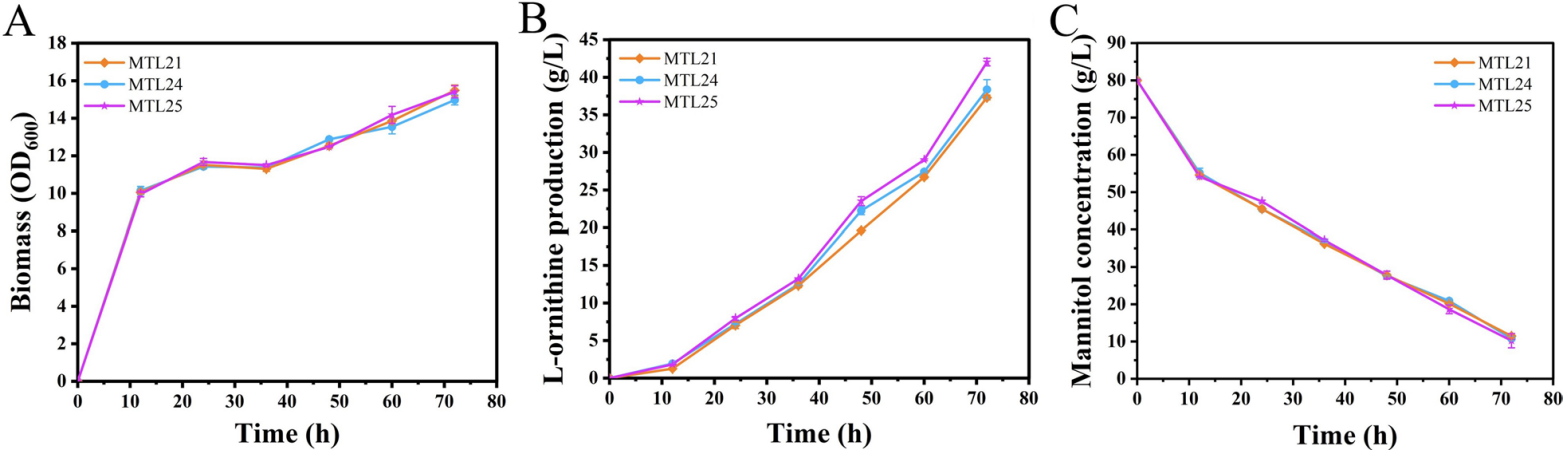
Effects of combining manipulate *qsuR*, *pdxR*, and *CGS9114_RS08985* on l-ornithine fermentation. Cell growth (**A**), l-ornithine concentration (**B**) and mannitol residue (**C**) of strains *C. glutamicum* MTL21, MTL24 and MTL25 in shake-flask fermentation. Results of standard deviations present in three individual experiments

From these results, it can be concluded that the combination of *qsuR* (encoding a LysR-type regulator QsuR) overexpression with *pdxR* (encoding a MocR-type regulator PdxR) inactivation or *CGS9114_RS08985* (encoding a hypothesis protein) overexpression is beneficial for l-ornithine biosynthesis. Since the engineered strain MTL24 exhibited the highest l-ornithine production titer, overexpression of *CGS9114_RS08985* (encoding a hypothesis protein) using the promoter P_*sod*_ insertion approach was introduced into MTL24 to generate strain MTL25. The shake-flask fermentation test with strain MTL25 indicated that 42.01 g/L of l-ornithine, representing a 12.7% and 4.4% increase as compared with MTL21 and MTL24, respectively, was produced during 72 h of cultivation (Fig. 5A and Table 4). MTL25 shares the same cell growth and mannitol consumption rate, further confirming that the manipulation of *CGS9114_RS08985* (encoding a hypothesis protein) was not toxic (Fig. 5B, C, Table 4). The yield of MTL25 reached 0.60 g/g mannitol, which was higher than that of MTL21 (0.54 g/g) and MTL24 (0.55 g/g) (Table 4). It is speculated that the interruption of *CGS9114_RS08985* (encoding a hypothesis protein) might block the biosynthesis of by-products and promote l-ornithine accumulation.

### Fed-batch fermentation of engineered *C. glutamicum* MTL25

**Fig. 6:**
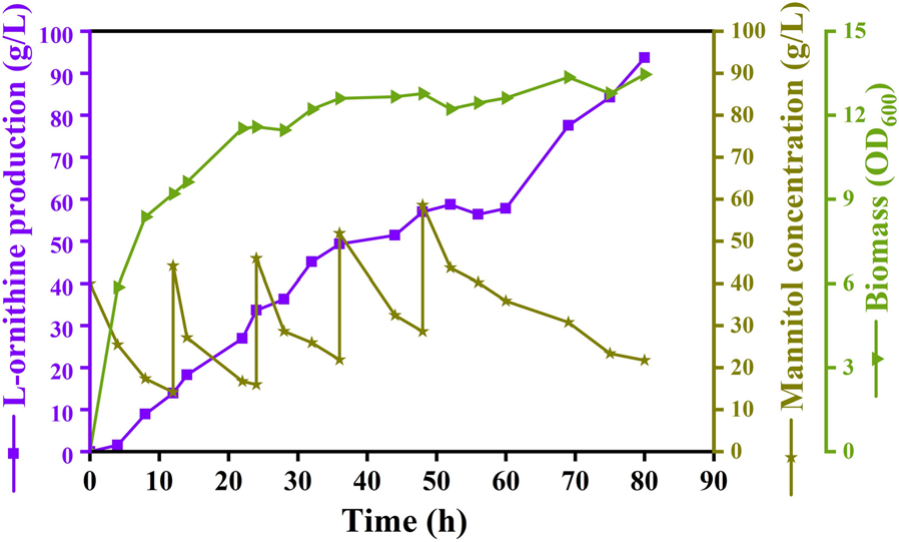
Fed-batch fermentation of the engineered strain MTL25 in 5-L fermenter. The purple, green, and deep yellow curves display trends of l-ornithine production, cell growth, and residue mannitol concentration, respectively

Fed-batch fermentation is generally used for performance evolution of engineered strains, which frequently produce higher titers of valuable compounds [36, 37]. For instance, *C. glutamicum* produced l-proline at a titer of 120.18 g/L [38]. In addition, fed-batch fermentation for 1,3-propanediol production was performed using the *C. glutamicum* strain MBP14, which produced 1,3-propanediol at a titer of 110.4 g/L [39]. Moreover, fed-batch fermentation was implemented for GABA production with a titer of 45.6 g/L GABA in a 7.5-L bioreactor [40]. Thus, the final strain MTL25 was scaled up in a 5-L bioreactor with fed-batch fermentation to further characterize l-ornithine production performance. During the fed-batch cultivation, the cell growth of MTL25 increased rapidly, and the OD_600_ reached 11.52 at 22 h (Fig. 6). The titer of l-ornithine produced by strain MTL25 reached 93.6 g/L at 80 h with a yield of 0.67 g/g mannitol, which was 123% higher than that obtained from shake-flask fermentation and represents the highest l-ornithine production titer (Fig. 6 and Table 5). The high conversion efficiency of mannitol to l-ornithine provides a robust foundation for higher utilization of macroalgae. In the past few decades, macroalgal biomass, a promising feedstock, has gained widespread interest from researchers for biofuel and bioproduct production [41]. High-value compounds have been produced using seaweeds and microorganisms. For instance, Lim et al. [42] constructed the engineered strain *Vibrio sp. Dhg* VDHG411 by overexpression of *pdc* and *aldB* from *Z. mobilis* and deletion of *ldhA*, *frdABCD*, and *pflB*, which produced ethanol at a titer of 19.2 g/L from kelp powder. Brown macroalgae, consisting of alginate, mannitol, fucoidan, and laminarin, are widely used in biorefinery processes. Engineered strain *C. glutamicum* SEA-3 was developed by the deletion of the mannitol repressor MtlR and heterologous expression of fructokinase and glyceraldehyde dehydrogenase that produced l-lysine at a yield of 0.24 mol/mol on mannitol [43]. This research group further obtained strain SEA-7 by introducing transhydrogenase PntAB and fructokinase Mak from *E. coli*, and *gapN* from *S. mutans*. The SEA-7 strain was expanded to produce l-lysine from *Laminaria digitata* extracts and *Durvillaea antarctica* waste stream with a yield of 0.27 and 0.4 mol/mol, respectively [44]. Recently, *C. glutamicum* CgRibo4, a riboflavin-producing strain, was generated via heterologous expression of *mtlDBs* and *mtlAFBs* and overexpression of the riboflavin operon *ribGCAH*. In fed-batch fermentation, this strain produced riboflavin at a titer of 1.29 g/L from *Laminaria hyperborea* extract [45]. Seaweed raw materials can be consumed and used for high-value compound production using engineered *C. glutamicum*. Compared with the strain in these studies, we developed a recombinant *C. glutamicum* MTL25 with superior l-ornithine production performance on mannitol by deletion of *mtlR*; overexpression of *mtlTD* operon, *pfkB*, *qsuR*, and *CGS9114_RS08985*; and attenuation of *pdxR*. If the genetic engineering breeding strategies developed in this study are applied to other *C. glutamicum*, the yield of lysine and riboflavin in brown macroalgae is expected to improve.

**Table 5:** Parameters of l-ornithine producing strain by genetic modification

Strains	l-Ornithine titer (g/L)	Yield (g/g)	Carbon source	Cultivation	Strategies	References
*C. glutamicum* MTL13	54.56	0.47	Mannitol	Fed-batch	Deletion of *mtlR*; overexpression of *mtlD* and *pfkB*	[21]
*C. glutamicum* KBJ11	88.26	0.414	Glucose	Fed-batch	Deletion of *argF*, *argR*, and *ncgl2228*; co-overexpression of *CsgapC* and *BsrocG*	[14]
*C. glutamicum* XAB03	41.5	ND	Xylose and glucose	Shake flask	Heterologous expression of *xylAB* (*X. campestris*) Overexpression of *pepc* and *araE*	[40]
*C. glutamicum* SO26	43.6	0.34	Glucose	Fed-batch	Deletion of *argF*, *ncgl1221*, *argR*, *putP*, *mscCG2*, and *iolR*; attenuation of *odhA*, *proB*, *ncgl22*28, *pta*, *cat*, and *pgi*; overexpression of *lysE*, *gdh*, *cg3035*, *pfkA*, *tkt*, *argCJBD*, *glt*, and *gdh2*	[13]
*C. glutamicum* SO29	18.6	0.40	Xylose	Shake flask	Heterologous expression of *xylAB* operon (*X. campestris*)	[13]
*C. crenatum* Cc-QF-4	12.6 ± 0.65; 40.4	0.126 ± 0.011; ND	Glucose	Shake flask; bioreactor	Deletion of *proB* and *argF*; co-expression of exogenous Ec *argA* and Sm *argE*	[6]
*C. glutamicum* 1006∆*argR*-*argJ*	31.6	0.396	Glucose	Shake flask	Deletion of *argR*; overexpression of *argJ*	[41]
*C. glutamicum* YW06 (pSY223)	51.5	0.240	Glucose	Fed-batch	Deletion of *proB*, *argR*, and *argF*; overexpression of *argCJBD*, *pgi*, *zwf*, and *tkt*;	[10]
*S. cerevisiae* M1dM2qM3e	5.1	ND	Glucose	Fed-batch	Modular pathway rewiring	[42]
*C. glutamicum* ∆APE6937R42(pEC-SacC)	22.0; 27.0	ND	Sucrose; molasses	Batch	Overexpression of *sacC (M. succiniciproducens)*	[16]
*C. glutamicum* SJC8260	12.48	ND	Glucose	Shake flask	Overexpression of *ncgl0462* and mutant *argCJBD*	[43]
*C. glutamicum* APE6937R42	24.1	0.298	Glucose	Bioreactor	Deletion of *argF*, *argR*, and *proB*; adaptive evolution in presence of l-ornithine	[4]
*C. glutamicum* SJC8399	13.16	ND	Glucose	Shake flask	Inactivation of *gntK*	[44]

## Conclusion

In this study, a transcriptome-guided genetic engineering approach was applied to the strain *C. glutamicum* MTL13, which promotes l-ornithine production from mannitol. Six genetic modulation targets obtained from transcriptome analysis were utilized to construct l-ornithine-producing strains that significantly facilitated the biosynthesis of l-ornithine from mannitol. We found that high yield and productivity of l-ornithine could be achieved by single overexpression of *qsuR* (encoding a LysR-type regulator QsuR) and *prpC* (encoding 2-methylcitrate synthase PrpC) or suppression of CGS9114_RS09730 (encoding a TetR/AcrR family transcriptional regulator). The genetic manipulation of a single gene can also be employed for the construction of engineered *C. glutamicum* strains to produce other glutamate family products. We also identified that the combined overexpression of *qsuR* (encoding a LysR-type regulator QsuR) and suppression of *CGS9114_RS09730* (encoding a TetR/AcrR family transcriptional regulator) exerted a superimposed acceleration effect on l-ornithine accumulation. By further overexpressing the hypothetical protein CGS9114_RS08985, the final strain MTL25 can produce 42.01 g/L l-ornithine during shake-flask fermentation. The fed-batch of *C. glutamicum* MTL25 was performed in a 5-L bioreactor for l-ornithine production, with a titer of 93.6 g/L and a yield of 0.67 g/g mannitol. The obtained titer and yield represent a 71.6% and 42.6% increase, respectively, as compared with the parent strain MTL13 (54.56 g/L and 0.47 g/g mannitol) [21]. These results illustrate that transcriptome analysis-guided rational genetic modification is a promising strategy for finding new indirect targets to improve strain performance. The ability to generate the highest l-ornithine production titer from mannitol will also accelerate the utilization of brown algae as a raw material to produce high-value-added chemicals.

## Methods

### Microorganisms, plasmids, and primers

The strains and plasmids used in the present study are listed in Table 6. These different l-ornithine-producing mutants were derived from *C. glutamicum* S9114 by electroporation and multiple rounds of selection with 12.5 μg/mL kanamycin and 10% sucrose for beneficial mutations that increase l-ornithine production. The strong promoter P_*sod*_ or T terminator was inserted into the upstream region of the target genes for gene overexpression and gene attenuation by virtue of the integration vector pK18*mobsacB* on the chromosome.

**Table 6:** Strains and plasmids used in this study

Strain or plasmid	Description	Source
*Strains*		
*E. coli* DH5α	Gene cloning of host bacteria	Transgen
MTL01	SO26 with deletion of mtlR	[21]
MTL13	l-Ornithine producing strain derived from *C. glutamicum* S9114	
MTL14	MTL13 with P_*sod*_ promoter inserted in the upstream region of *qsuR*	This study
MTL15	MTL13 with P_*sod*_ promoter inserted in the upstream region of *prpC*	This study
MTL16	MTL13 with P_*sod*_ promoter inserted in the upstream region of *CGS9114_RS08985*	This study
MTL17	MTL13 with terminator inserted in the upstream region of *acnR*	This study
MTL18	MTL13 with terminator inserted in the upstream region of *CGS9114_RS09730*	This study
MTL19	MTL13 with terminator inserted in the upstream region of *pdxR*	This study
MTL20	MTL14 with P_*sod*_ promoter inserted in the upstream region of *prpC*	This study
MTL21	MTL14 with P_*sod*_ promoter inserted in the upstream region of *CGS9114_RS08985*	This study
MTL22	MTL14 with terminator inserted in the upstream region of *acnR*	This study
MTL23	MTL14 with terminator inserted in the upstream region of *CGS9114_RS09730*	This study
MTL24	MTL14 with terminator inserted in the upstream region of *pdxR*	This study
MTL25	MTL24 with P_*sod*_ promoter inserted in the upstream region of *CGS9114_RS08985*	This study
*Plasmid*		
pK18*mobsacB*	Mobilizable vector, allows for selection of double crossover in *C. glutamicum*, Km^R^, *sacB*	Laboratory stock
pK18-T- *CGS9114_RS09730*	A derivative of pK18*mobsacB*, harboring T- *CGS9114_RS09730* fragment	This study
pK18-T-*pdxR*	A derivative of pK18*mobsacB*, harboring T- *pdxR* fragment	This study
pK18-T-*acnR*	A derivative of pK18*mobsacB*, harboring T-*acnR* fragment	This study
pK18-P_*sod*_-*qsuR*	A derivative of pK18*mobsacB*, harboring P_*sod*_-*qsuR* fragment	This study
pK18-P_*sod*_-*prpC*	A derivative of pK18*mobsacB*, harboring P_*sod*_-*prpC* fragment	This study
pK18-P_*sod*_- *CGS9114_RS08985*	A derivative of pK18*mobsacB*, harboring P_*sod*_-*CGS9114_RS08985* fragment	This study

The plasmid construction procedure was as follows. The homologous arms of the target genes, including *qsuR*, *prpC*, *pdxR*, *acnR*, *CGS9114_RS08985*, and *CGS9114_RS09730,* were amplified by polymerase chain reaction (PCR), and the primers were designed based on the published *C. glutamicum* S9114 genome sequence (Additional file 1: Table S1). PCR products were cloned into pK18 using a one-step PCR cloning kit (Novoprotein). LB medium supplemented with one part per thousand kanamycin (50 μg/mL) was used as the standard medium for plasmid construction in *E. coli*. The plasmids were transformed into *C. glutamicum* MTL13-competent cells by electroporation (3000 V, 4 ms).

### Growth conditions

For transcriptome analysis, MTL01 was inoculated from seed medium cultured in exponential growth period fermentation medium with 80 g/L glucose and 80 g/L mannitol at 32 °C and 250 rpm, respectively. The shake-flask test was performed as described previously [7, 8, 12] and all flask cultures were repeated three times. For the scale-up of l-ornithine production, fed-batch fermentation was performed in a 5-L fermenter (BIOTECH-5JG, Bao Xing, China), which was equipped with a toothed defoaming paddle with an outer diameter of 95 mm and a six-bladed impeller with an outer diameter of 90 mm placed in the agitator shaft, which was distributed at 20 and 3 cm from the bottom, respectively. After resuscitating in agar plates, two rings of strain pellets were inoculated into a 150-mL shake-flask containing 13 mL of LBG medium (Luria–Bertani broth supplemented with 20 g/L glucose) at 32 °C with 250 rpm for 12 h. The seed cultures were prepared in 30-mL shake flasks at 32 °C with 250 rpm and 5 mL of LBG culture. The seed medium contained 30 g/L glucose, 10 g/L yeast extract, 10 g/L corn steep liquor, 15 g/L (NH_4_)_2_SO_4_, 2.5 g/L anhydrous MgSO_4_, 1 g/L KH_2_PO_4_, 0.5 g/L K_2_HPO_4_, 0.5 g Na_2_HPO_4_, and 10 g/L CaCO_3_. After 12 h of cultivation, the OD_600_ reached approximately 8.5, and 225 mL of the seed culture was transferred into a 5-L bioreactor equipped with dissolved oxygen (DO), temperature, and pH control units and 2 L fermentation medium. The initial medium fermentation medium contained 40 g/L mannitol, 6 g/L yeast extract, 50 g/L (NH_4_)_2_SO_4_, 2.5 g/L anhydrous MgSO_4_, 1 g/L KH_2_PO_4_, 0.5 g/L K_2_HPO_4_, 0.5 g/L Na_2_HPO_4_, 0.02 g/L MnSO_4_⋅H_2_O, and 0.02 g/L FeSO_4_⋅7H_2_O. After inoculation for 12 h, the feeding solution was continued for 12 h to maintain a residual mannitol concentration above 10 g/L from 12 to 48 h. The feed solution contained 450 g/L mannitol, 5 g/L yeast extract, 5 g/L (NH_4_)_2_SO_4_, and 1 g/L anhydrous MgSO_4_. Cultivation was sustained for 72 h with an air mass flow of 2 L/min and a temperature of 32 ℃. The pH, DO, and temperature were monitored in real time and automatically adjusted during the entire process. DO was maintained at 30% by adjusting the agitation speed. The pH was controlled at 6.90 with 25% (v/v) ammonia solution and manually added defoamer propoxylated glycerin (Hangzhou Pursue Biotechnology Co, LTD, China), if necessary. The dynamic curves of the stirrer speed, temperature, dissolved oxygen, and pH during the fermentation process are depicted in Additional file 1: Fig. S1.

### RNA-seq and transcriptome analysis

Total RNA was extracted from 25 mL of culture (collected at 12 h, OD_600_ 9.5) using TRIzol ® Reagent (Invitrogen), and genomic DNA was removed with DNase I (TaKara). RNA quality and quantity of RNA was tested using a Bioanalyzer 2100 (Agilent) and ND-2000 (NanoDrop Technology), respectively. The cDNA library was constructed by performing mRNA purification using the Ribo-Zero Magnetic kit (Epicenter), fragmentation of mRNA by UNG enzyme, cDNA synthesis, adapter connection by End Repair Mix, and amplification by Phusion DNA polymerase, one after another according to the manufacturer’s instructions. The paired-end RNA-seq sequenced and clean data were obtained by removing reads with low sequencing quality and adapter sequences on the Illumina platform. Raw reads of our transcriptome data are deposited in the NCBI Short Read Archive under the accession number PRJNA859005. Bioinformatics analysis was performed on the Majorbio Cloud platform (www.majorbio.com) based on the Illumina platform-generated data. High-quality reads were mapped to the *C. glutamicum* S9114 genome (NZ_AFYA01000001.1) using the Bowtie 2. The TPM (transcripts per kilobase million) value and fragments per kilobase per million reads (FPKM) were counted separately for each gene by Kallisto and Salmon, which were used to measure expression levels. Differential expression analysis was performed using the software package DESeq2 with *p*-adjust < 0.05 and |log2FC|≥ 1. Goatools were applied for GO enrichment analysis using Fisher’s exact test and discovery rate for *p*-adjust. KEGG enrichment analysis was established using KOBA, and the method of *p*-adjustment was the same as that used for GO.

### Cell growth and metabolite concentration measurement

Cell growth was determined by measuring the OD_600_ value of the culture solution using a microplate reader (Waters Instruments, MA, USA). The concentration of residual mannitol was represented by OD_412_ using a color reaction [21, 46]. Then, 100 μL of sample dilution was successively mixed with 100 μL of 0.015 M NaIO_4_ solution for 10 min, 200 μL of 0.1% l-rhamnose solution, and 400 μL of Nash reagent (150 g/L ammonium acetate, 2 mL/L acetic acid, and 2 mL/L acetylacetone). The mixture was then bathed in 53 °C water for 15 min and scanned using a microplate reader at a wavelength of 412 nm. l-Ornithine concentrations were measured according to a previous report [47].

## Supplementary Information

**Additional file 1: Table S1.** Primers used in this study. **Table S2.** Promoter and terminator sequence used in this study. **Figure S1.** The dynamic curve of stirrer speed, temperature, dissolved oxygen, and pH overall fermentation process.

## Data Availability

Data have been deposited in the NCBI Short Read Archive under accession number PRJNA859005.

## Abbreviations

LB: Luria–Bertani
NADPH: Nicotinamide adenine dinucleotide phosphate
