# Supplementary material for: Improvement in l-ornithine production from mannitol via transcriptome-guided genetic engineering in Corynebacterium glutamicum
Source: Biotechnol Biofuels Bioprod. 2022 Sep 19;15:97. doi: 10.1186/s13068-022-02198-8 (PMC9484086; doi:10.1186/s13068-022-02198-8)
Supplement: Supplementary file 1 — Additional file 1: Table S1. Primers used in this study. Table S2. Promoter and terminator sequence used in this study. Figure S1. The dynamic curve of stirrer speed, temperature, dissolved oxygen, and pH overall fermentation process. [file 13068_2022_2198_MOESM1_ESM.docx]

**Table S1** Primers used in this study.

| Primer | Sequence and purpose (5'–3') |
| --- | --- |
| T-check-F | GGCGGTCAGATGATCGCCCTT |
| *CGS9114_RS09730*-up-F | aacgacggccagtgccaagct GTGATGTTCACGATCGTTCCC |
| *CGS9114_RS09730*-up-R | AAAAGGGCGATCATCTGACCGCCCCAACCTTCGCCACATGTTC |
| *CGS9114_RS09730*-down-F | GGGCGGTCAGATGATCGCCCTTTTTTTTTTTCGTCAGCGAATAACCATGC |
| *CGS9114_RS09730*-down-R | cggtacccggggatcctctag TCCGTCATCGCGTTGATC |
| *acnR*-up-F | aacgacggccagtgccaagctTGCTTTCGGCATCATCGAC |
| *acnR*-up-R | AAAAGGGCGATCATCTGACCGCCCTTGCACGCTTGTAGGAAACTCG |
| *acnR*-down-F | AAGGGCGGTCAGATGATCGCCCTTTTTTTTTTGGTGATCAACCAATTGCGG |
| *acnR*-down-R | cggtacccggggatcctctag AACGCGCCGATTGATGAG |
| *pdxR* -up-F | aacgacggccagtgccaagct TTTCGCAGCTTCCAACCAG |
| *pdxR* -up-R | AAAAGGGCGATCATCTGACCGCCC AATGCCGGAATTCTTGCAC |
| *pdxR* -down-F | GGGCGGTCAGATGATCGCCCTTTTTTTTTTATCCAGTCGTGTCGGTCTACC |
| *pdxR* -down-R | cggtacccggggatcctctag AAGCGATGTTTACGTGCATCC |
| P_sod_-F | GCTGCCAATTATTCCGGGCTTG |
| *qsuR* -up-F | aacgacggccagtgccaagct CCAACCCCATGACATTAGAGC |
| *qsuR* - up-R | CGGAATAATTGGCA AGGGTCATACATCCACCCCTT |
| *qsuR* -P_sod_-F | TGTATGACCCT TGCCAATTATTCCGGGCTTG |
| *qsuR* -P_sod_-R | GGTATGCAAGAGATGGATATCATGGTTTCCGCACCGAGCATATACATCTT |
| *qsuR* -down-F | CCATGATATCCATCTCTTGCATACC |
| *qsuR* -down-R | cggtacccggggatcctctagTTCTGGATCAGGGCCTTCAC |
| *prpC*-up-F | aacgacggccagtgccaagct GTCGACAAGCATTTAAAGCGG |
| *prpC* - up-R | CGGAATAATTGGCA CGCAGGAGCTCATATAATCTGC |
| *prpC* -P_sod_-F | GAGCTCCTGCG TGCCAATTATTCCGGGCTTG |
| *prpC* -P_sod_-R | ATGTGCTGGTCAAAGACGTTGTATTTCCGCACCGAGCATATACATCTT |
| *prpC* -down-F | TACAACGTCTTTGACCAGCACAT |
| prpC -down-R | cggtacccggggatcctctag TTACGATCCAACGCCTTGC |
| *CGS9114_RS08985*-up-F | aacgacggccagtgccaagctTGACCGTCCTATGTGACCGAT |
| *CGS9114_RS08985*-up-R | CGGAATAATTGGCATAAGCGCAGTTCAGTAGGGG |
| *CGS9114_RS08985*5-P_sod_-F | AACTGCGCTTA TGCCAATTATTCCGGGCTTG |
| *CGS9114_RS08985*-P_sod_-R | CTGGTTTCCAAGCATAGGCATTTCCGCACCGAGCATATACATCTT |
| *CGS9114_RS08985*-down-F | TGCCTATGCTTGGAAACCAG |
| *CGS9114_RS08985*-down-R | cggtacccggggatcctctagTCGCGATATACGCTGCTGC |

**Table S2** Promoter and terminator sequence used in this study.

| Name | Sequence (5'–3') |
| --- | --- |
| P_sod_ | AGCTGCCAATTATTCCGGGCTTGTGACCCGCTACCCGATAAATAGGTC  GGCTGAAAAATTTCGTTGCAATATCAACAAAAAGGCCTATCATTGGGA  GGTGTCGCACCAAGTACTTTTGCGAAGCGCCATCTGACGGATTTTCAAA  AGATGTATATGCTCGGTGCGGAAA |
| T | GGGCGGTCAGATGATCGCCCTTTTTTTTTT |


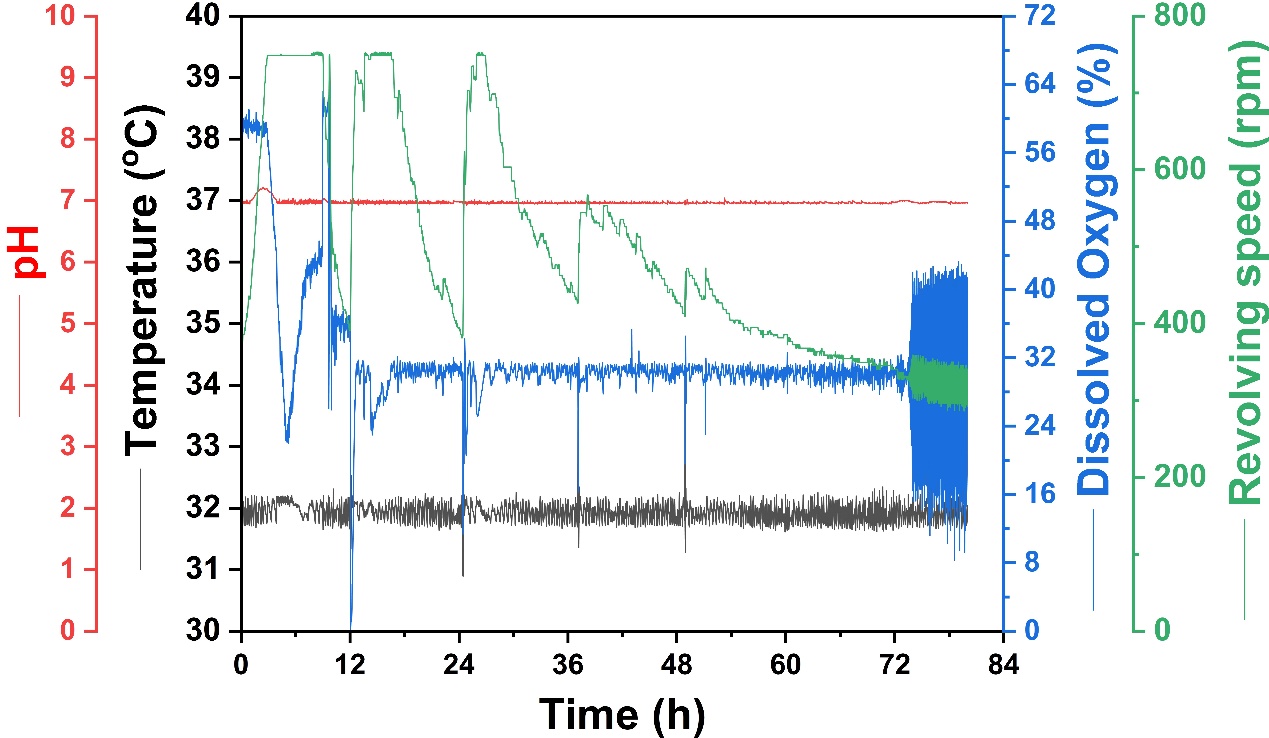


Figure S1 The dynamic curve of stirrer speed, temperature, dissolved oxygen, and pH overall fermentation process. The red, black, blue, and green curves represent pH, temperature, dissolved oxygen, and stirrer speed respectively.
